# Bacterial persisters are a stochastically formed subpopulation of low-energy cells

**DOI:** 10.1371/journal.pbio.3001194

**Published:** 2021-04-19

**Authors:** Sylvie Manuse, Yue Shan, Silvia J. Canas-Duarte, Somenath Bakshi, Wei-Sheng Sun, Hirotada Mori, Johan Paulsson, Kim Lewis

**Affiliations:** 1 Antimicrobial Discovery Center, Department of Biology, Northeastern University, Boston, Massachusetts, United States of America; 2 Department of Systems Biology, Harvard Medical School, Boston, Massachusetts, United States of America; 3 Graduate School of Biological Sciences, Nara Institute of Science and Technology, Nara, Japan; Universitat zu Koln, GERMANY

## Abstract

Persisters represent a small subpopulation of non- or slow-growing bacterial cells that are tolerant to killing by antibiotics. Despite their prominent role in the recalcitrance of chronic infections to antibiotic therapy, the mechanism of their formation has remained elusive. We show that sorted cells of *Escherichia coli* with low levels of energy-generating enzymes are better able to survive antibiotic killing. Using microfluidics time-lapse microscopy and a fluorescent reporter for in vivo ATP measurements, we find that a subpopulation of cells with a low level of ATP survives killing by ampicillin. We propose that these low ATP cells are formed stochastically as a result of fluctuations in the abundance of energy-generating components. These findings point to a general “low energy” mechanism of persister formation.

## Abstract 2

Persisters represent a small subpopulation of non- or slow-growing bacterial cells that are tolerant to killing by antibiotics, but the mechanism of their formation has remained elusive. This study of E. coli shows that stochastic heterogeneity in levels of energy-generating enzymes results in a subpopulation of cells with low ATP that are tolerant to antibiotics.

## Main text

In *Escherichia coli*, the RNA-degrading interferase-type toxin/antitoxins (TA) emerged as the main component involved in persister formation [1]. These findings, however, have recently been challenged [2–5]. At the same time, several TAs from other classes have been firmly linked to persisters in *E*. *coli* [6]. The HipA toxin is a glutamyl-tRNA kinase [7,8], and its gain-of-function mutants produce high levels of persisters by inhibiting translation in patients with relapsing urinary tract infection [9] while its absence leads to a decrease in the level of persisters in *Caulobacter crescentus* [10]. The SOS response induces TisB toxin which forms a pore in the membrane [11], decreasing the proton motive force and ATP, leading to persister formation [12,13]; the HokB toxin acts in a similar way [14,15]. However, the mechanism by which persisters form over the normal course of growth remains unclear. Different studies suggested slow growth/inhibition of translation [16]; drop in proton motive force [17]; ppGpp [18]; as the main component responsible for persister formation. Currently, there is no agreement on the underlying cause of persister formation and drug tolerance.

Previously, we suggested that a drop in ATP may represent such a mechanism [2,3,19]. Stationary phase cells have lower ATP levels, and we found that persisters in a growing population of *Staphylococcus aureus* have elevated levels of expression of stationary phase markers, suggesting that those cells would also have a lower level of ATP [2]. We made similar observations in *E*. *coli* [3], and we recently showed that fluctuations in the expression of energy-generating Krebs cycle enzymes are linked to persisters in *S*. *aureus* [20].

**Fig 1:**
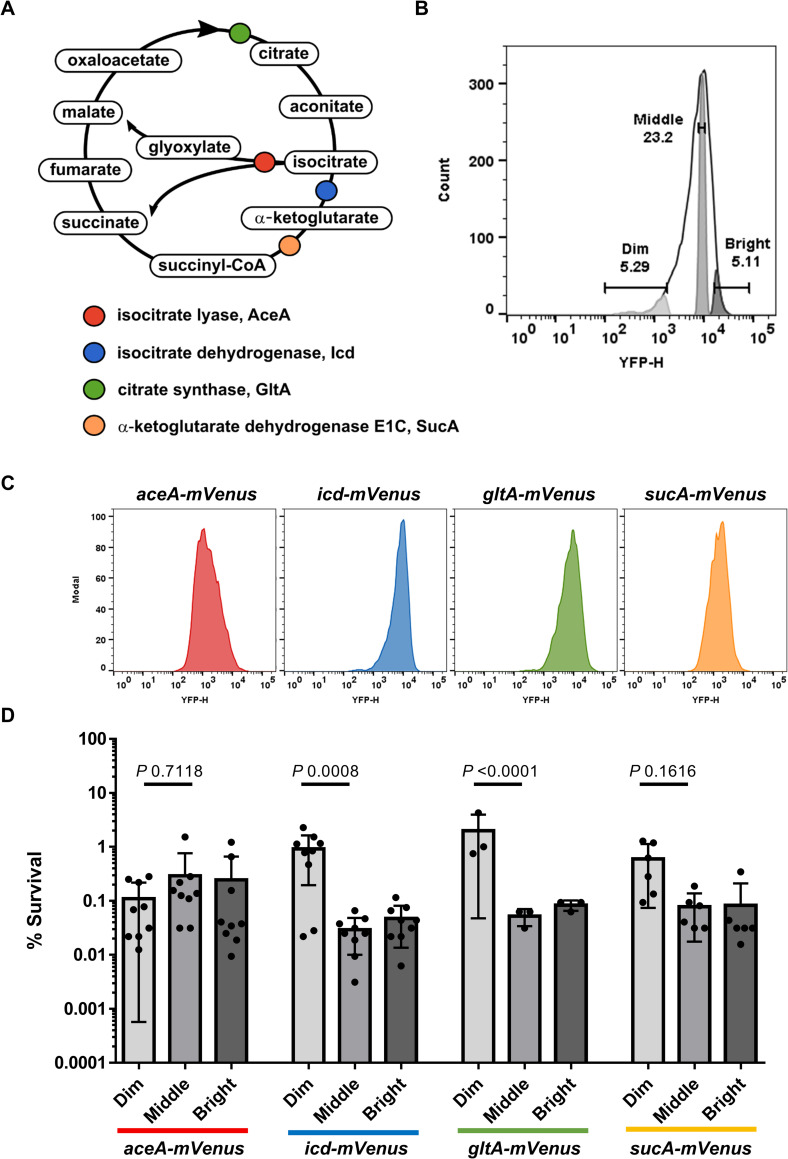
*E*. *coli* populations with low abundance of energy-producing enzymes are enriched in persisters. (A) Schematic of the Krebs cycle reactions. Tested enzymes have been color coded. (B) Representative peak of fluorescence of the different energy-producing enzyme reporters tested in this study. For each reporter, Dim (approximately 5%), Middle (approximately 20%), and Bright (approximately 5%) population gates were created based on mVenus signal recorded from a YFP detector. (C) Representative fluorescent peaks of each reporter before cell sorting. The number of events represented in the *y* axis is normalized for each graph. (D) Exponentially growing cells of *E*. *coli* W3110D carrying a translational mVenus fusion of *aceA*, *icd*, *gltA*, *sucA* (grown in LB at 37°C) were exposed to 1 μg/mL of ciprofloxacin for 4 hours. The antibiotic treated cells were then analyzed by FACS as shown in (C). Dim, Middle, and Bright fractions were isolated by cell sorting as shown in (B) onto LB agar. The percentage survivals of the Dim, Middle, and Bright fractions correspond to the comparison between the CFU counts and the total numbers of sorted cells. Data are the average results from at least 3 biological replicates (*n* = 9, 9, 3, 6, for *aceA-mVenus*, *icd-mVenus*, *gltA-mVenus*, *sucA-mVenus*, respectively). Error bars represent standard deviations. Significance was determined using two-way ANOVA and Tukey’s multiple comparison tests. The underlying data for this figure can be found in S1 Data. CFU, colony forming unit; FACS, fluorescence-activated cell sorting; YFP, yellow fluorescent protein.

We therefore first sought to examine if impaired Krebs cycle activity would also impact persister formation in an unrelated microorganism, *E*. *coli*. Isocitrate dehydrogenase (Icd) catalyzes the rate-limiting step of the Krebs cycle (Fig 1A), and it has been shown that an *icd* mutant produces less ATP [21]. We observed that an *icd* mutant produced more persisters tolerant to killing by ciprofloxacin (S1A Fig) and confirmed that it had a lower level of ATP (S1B and S1C Fig). By contrast, the persister level did not change in a mutant of isocitrate lyase (AceA) which uses the same substrate as Icd but is not part of the canonical Krebs cycle; it catalyzes the first step of the glyoxylate shunt (Fig 1A). Only Icd will contribute to the generation of ATP in LB medium. The *icd* mutant had a modest growth defect (S2A Fig). The results suggest that under those experimental conditions, the Krebs cycle but not the glyoxylate shunt is involved in persister formation.

We then examined whether impaired Krebs cycle activity in single cells of a wild type (WT) population would lead to persister formation. For this, we constructed chromosomal mVenus translational fusions replacing native genes coding for key Krebs cycle enzymes: GltA (citrate synthase), Icd (isocitrate dehydrogenase), and SucA (α-ketoglutarate dehydrogenase) (Fig 1A). As a control, we also made a fusion of the glyoxylate shunt enzyme AceA which uses the same substrate as Icd but is not part of the canonical Krebs cycle and, importantly, does not play a role in the cell metabolism during growth in rich medium but only with acetate or fatty acids as sole carbon sources [22]. In the presence of pyruvate as the sole carbon source, a functional Krebs cycle is essential for growth (S2B Fig), while with acetate as the sole carbon source, *aceA* becomes essential (S2C Fig). We used those conditions to test the functionality of the mVenus fusions (S2B and S2C Fig). We observed that the Krebs cycle fusions grow similarly as the WT in LB and in minimal medium + pyruvate (S2A and S2B Fig). The *aceA-mVenus* is functional as well, though cells are growing somewhat slower than the WT in minimal medium + acetate (S2C Fig). The mVenus fusion strains were grown to exponential phase and challenged with a lethal dose of ciprofloxacin. The fluorescence of the cultures was measured by fluorescence-activated cell sorting (FACS) and showed in all cases a wide range of variation in the abundance of Krebs cycle enzymes among cells (Fig 1C). Interestingly, after the addition of ciprofloxacin, there was a shift to higher fluorescence levels, and an increase in heterogeneity of the population (S3A and S3B Fig). Bright, Middle, and Dim cells were sorted onto LB agar plates (Fig 1B) and survival was assessed (Fig 1D). The purity of sorted cells was also confirmed by a post-sorting purity analysis (S3C–S3F Fig). The survival of Dim populations with low levels of GltA, Icd, and SucA was higher as compared to the Middle populations, showing enrichment for persisters in cells with a diminished Krebs cycle expression (Fig 1D). There was no relationship between the levels of AceA and survival (Fig 1D), showing that a decrease in the level of the energy-producing Krebs cycle enzymes was specifically linked to persister formation.

**Fig 2:**
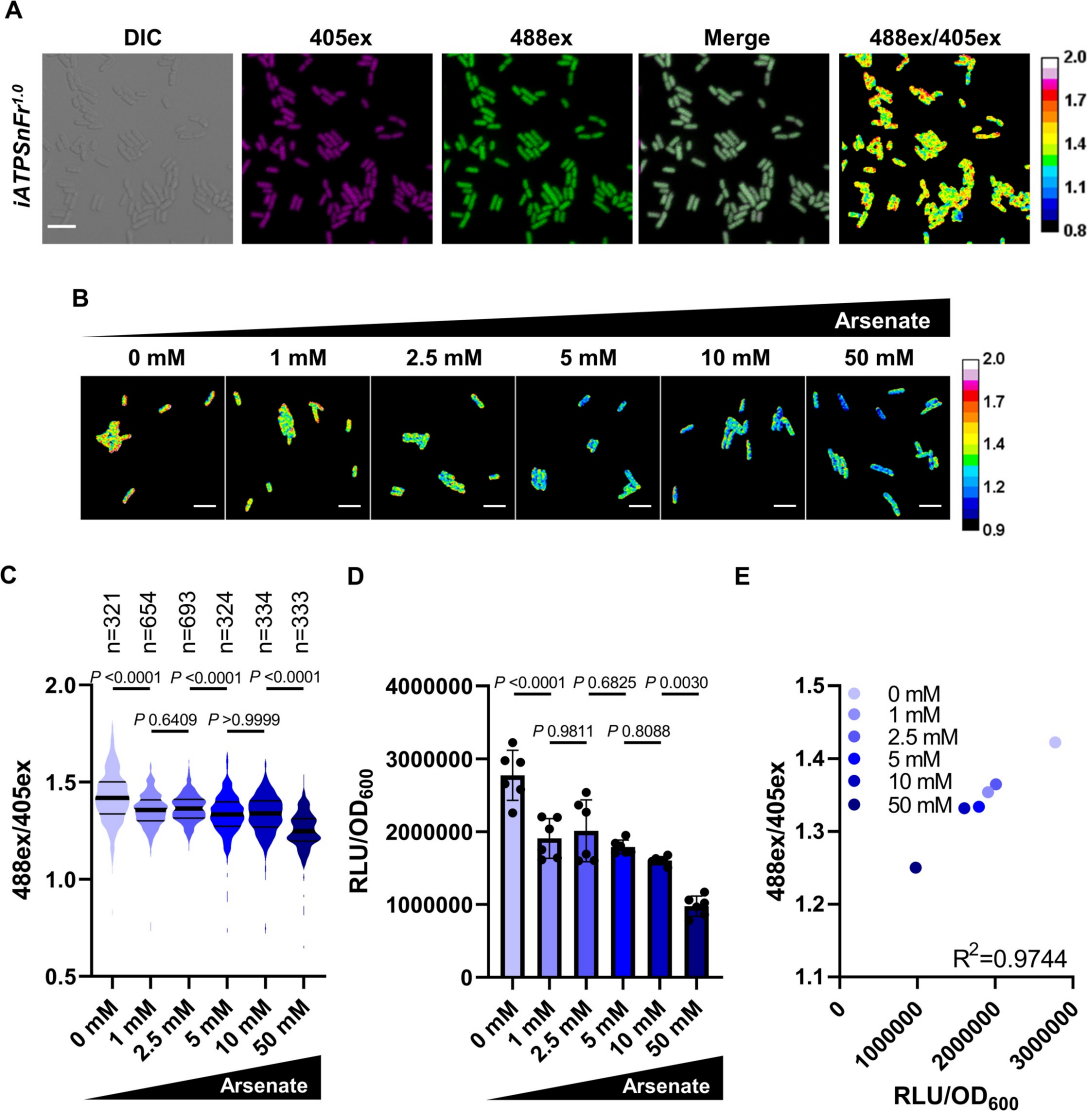
Expression and calibration of the ATP reporter iATPSnFr^1.0^ in *E*. *coli*. (A) Representative images of an exponential culture of MG1655_*iATPSnFr*^*1*.*0*^ cultured in LB at 37°C. The fluorescent signals from 405ex and 488ex are false colored in magenta and green, respectively. In the ratiometric 488ex/405ex panel, orange/yellow cells correspond to cells with higher ATP, and blue cells with lower ATP. Scale bar, 5 μm. Observations were performed with a confocal microscope. (B) Representative ratiometric 488ex/405ex images of MG1655-SB1_*iATPSnFr*^*1*.*0*^ exponential cultures treated 30 minutes with different concentrations of arsenate. Scale bar, 5 μm. (C) Distribution of the iATPSnFr^1.0^ 488ex/405ex ratio per single cell in exponential cultures treated with arsenate. Thick lanes represent the median, and secondary lanes the quartiles. Significance was determined using Kruskal–Wallis and Dunn’s multiple comparison tests. Data are representative of experiments made twice giving similar results. (D) Bulk ATP levels were measured in MG1655-SB1_*iATPSnFr*^*1*.*0*^ grown and treated with arsenate as in (B) and (C) with firefly luciferase assay and normalized with OD_600_. Data are the average results from 2 independent experiments performed with 3 biological replicates (*n =* 6). Error bars represent standard deviations. Significance was determined using one-way ANOVA and Tukey’s multiple comparison tests. (E) Calibration curve showing the correlation between single-cell and bulk measurements of ATP done in (C) and (D). Each data point represents the average of both single-cell and bulk measurements. Correlation was examined by Pearson’s correlation. The underlying data for this figure can be found in S1 Data.

We then sought to measure ATP directly at the single-cell level in a WT population and examine the level of ATP in individual persisters. In order to detect ATP in single cells, we evaluated different ratiometric ATP sensors [23,24]. The QUEEN reporter [23] had a low level of fluorescence when expressed from the chromosome, and increasing the copy number through plasmid expression led to toxicity. We found that the QUEEN derivative iATPSnFr^1.0^ [24] works well in *E*. *coli*. This reporter is composed of a circularly permuted superfolder GFP and an ATP-binding subunit of *Bacillus PS*3 F_0_F_1_ ATP synthase. iATPSnFr^1.0^ absorbs at 2 different wavelengths (405 nm and 488 nm) and emits at 515 nm. Binding to ATP increases the fluorescence from the 488 nm excitation but does not significantly change the fluorescence from the 405 nm excitation [24]. A ratio between signals from the 2 excitation wavelengths is calculated to report ATP concentration (488ex/405ex ratio) and is self-normalized for variation in iATPSnFr^1.0^ levels among cells, which is critical for accurate single-cell analysis. We expressed iATPSnFr^1.0^ in *E*. *coli* from the chromosome under a constitutive promoter. This sensor was originally designed for use in neurons, but we observed good fluorescence intensity in *E*. *coli* as well (Fig 2A) without affecting growth (S2D Fig). To test the ability of the sensor to report ATP changes in *E*. *coli*, we applied increasing concentrations of arsenate in order to deplete ATP [3] in an exponential culture of *E*. *coli* expressing iATPSnFr^1.0^. The fluorescence intensities of individual cells were recorded by microscopy (Fig 2B), and the 488ex/405ex ratio in cultures with different concentrations of arsenate was analyzed (Fig 2C). We observed that the distribution was significantly shifted towards a lower 488ex/405ex ratio after arsenate treatment. This is consistent with a decreased ATP level. Qualitatively, this is evident by the shift in artificial color from yellow/orange to blue cells (Fig 2B). We performed the same experiment by measuring ATP in the bulk of the population using the firefly luciferase assay (Fig 2D) and compared it to single-cell levels of ATP (Fig 2E). We observed a strong correlation between the 2 values showing that iATPSnFr^1.0^ is indeed reporting ATP in *E*. *coli*. We also compared single-cell and bulk measurements of ATP between a stationary phase and an exponential culture of *E*. *coli* and observed lower ATP levels in stationary phase and higher in an exponential culture (S4A–S4C Fig). In both cultures, heterogeneity in ATP levels was observed (S4B Fig). However, despite these encouraging validations, we do not draw conclusions based on quantitative values, but rather consider relative measurements between subpopulations in pooled mixtures of cells.

**Fig 3:**
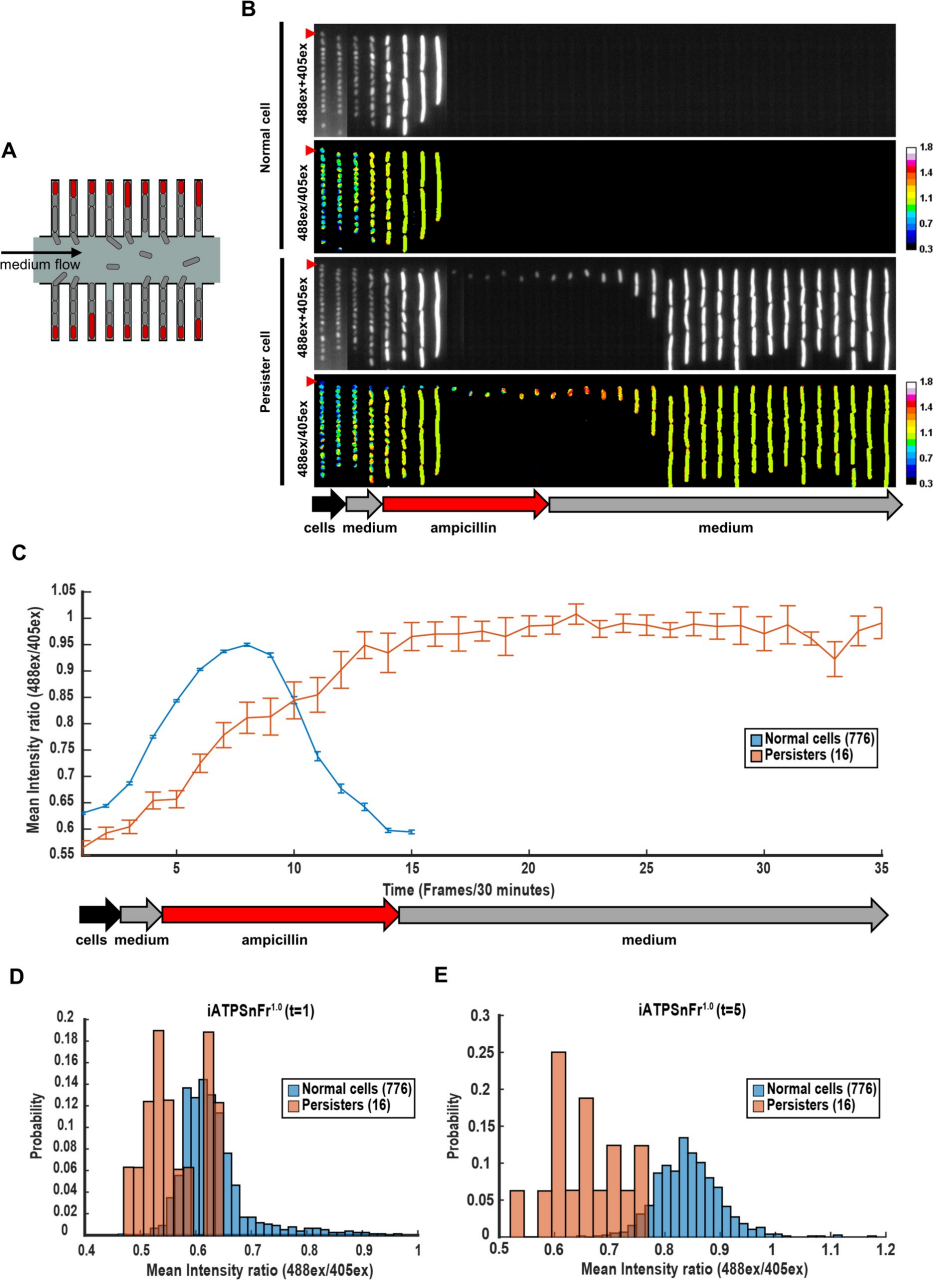
Persisters have lower ATP level than the rest of the population. (A) Schematic of the mother machine device. Only mother cells (in red) at the end of each cell channel were tracked. (B) Kymographs of 2 single channels with MG1655-SB1_*iATPSnFr*^*1*.*0*^ cells over time. Stationary phase cells were concentrated and loaded in the device. The culture used to load the device was first flowed in the device for 1 hour (black arrow), followed by fresh EZRDM for 1 hour (grey arrow), by EZRDM + ampicillin (50 μg/mL) for 5 hours (red arrow), and finally by fresh EZRDM again (grey arrow). Frames were taken 30 minutes apart. The two upper panels represent the outcome of a mother cell that dies following ampicillin treatment. The two lower panels represent the outcome of a persister cell that survives ampicillin treatment. The 488ex+405ex panels represent the addition of iATPSnFr^1.0^ 405ex and 488ex intensities. The iATPSnFr^1.0^ ratiometric 488ex/405ex panels represent ATP level. The mother cells analyzed are indicated with a red triangle. Observations were performed with a time-lapse epifluorescence microscope. (C) Data represented are the mean of the iATPSnFr^1.0^ 488ex/405ex ratio per single cell over time frames (frames interval is 30 minutes). Error bars represent standard errors. (D) Distribution of the iATPSnFr^1.0^ 488ex/405ex ratio per single cell for normal and persister cells at the first time frame of the time lapse (t = 1), and (E) immediately before the antibiotic is added (t = 5). The underlying data for this figure can be found in S1 Data.

We reasoned that the low ATP cells detected by the iATPSnFr^1.0^ reporter could be persisters that survive antibiotic treatment. Persisters make up a small part of the population, and in order to track a reasonable number of these cells, we employed microfluidics time-lapse microscopy in a mother machine device [25]. In these devices, bacteria are loaded into growth trenches that are orthogonal to a media channel. The bacteria positioned at the dead end of the trenches are referred to as “mother cells” and are kept throughout the experiment as progenitors of a linearly growing population (Fig 3A), while some descendent cells are continuously pushed out of the trenches. To increase the number of persister cells observed, we loaded an aged stationary phase culture (48 hours post-inoculation) [26] of cells expressing iATPSnFr^1.0^ (Fig 3B) in a high-throughput version of the mother machine [27]. In order to preserve the growth conditions of the test tube, we flowed the same culture used to load the mother machine for 1 hour through the device. Fresh medium was then flowed for 1 hour, and cells began to increase their ATP level and started to grow (Fig 3B and 3C). Fresh medium containing ampicillin was then flooded into the device for 5 hours. In total, 90,952 individual lineages were scanned. About 5% to 10% of cells did not grow and divide before or after drug (S5 Fig), in agreement with previous reports [28–30] and likely correspond to dead cells present in stationary populations. The majority of cells instead elongated and died during antibiotic treatment, except for 2 cells that elongated and eventually survived, most likely because the ampicillin exposure was relatively brief (S5 Fig). Approximately 1% to 2% of cells elongated in the presence of ampicillin without lysing but failed to regrow after the antibiotic was washed away. These cases represent death without lysis (S5 Fig). We then observed 16 cells that survived the antibiotic and were able to restart growth after ampicillin was replaced with fresh medium, producing a viable lineage (survival rate of 0.018%) (Fig 3B, S6A Fig, and S1 Movie). All except one did not grow prior to the addition of the antibiotic, and the last showed an atypically slow growth before addition of ampicillin (S6A Fig). These persisters resumed growth heterogeneously after an extended lag phase (S6B Fig), corroborating the pioneering work of Balaban and colleagues [31] as well as other studies [32–34].

Because this set-up allows us to track cells for long time periods before, during, and after the antibiotic treatment, we next analyzed ATP levels of the 16 individual persisters and 776 regular cells over the course of the experiment. Strikingly, persisters have lower ATP both at the onset of wake up from stationary phase and at the start of antibiotic treatment (Fig 3C–3E and S7A–S7C Fig). To test for statistical significance, we randomly sampled 16 of the 776 regular cells and calculated the average estimated ATP levels, repeating this computation 100,000 times. In no case did we observe an average ATP level as low or lower than that of the 16 persister cells, showing that the effect is highly significant statistically. In addition, persisters more gradually increased their ATP level prior to division compared to the cells that later lysed (Fig 3C). These results are consistent with protein aggregation at low ATP level favoring persister formation; an increase in ATP enables disaggregation and resumption of growth [30]. Interestingly, we also observed that persisters are on average smaller compared to the other cells (S8A Fig). This is consistent with what we recently reported [27]. This suggests that both low ATP level and small cell size can predict the ability to survive antibiotics. Interestingly, we found no correlation between levels of ATP and cell size in the rest of the population (S8B Fig). Single-cell isolation from mother machine devices followed up by metabolomics analysis will allow to independently validate in the future that persisters have lower ATP levels and will allow as well to highlight other key features of persisters [35].

We previously reported that depleting ATP by treating cells with arsenate inhibits antibiotic targets such as DNA gyrase/topoisomerase and the ribosome, correlating with an increase in antibiotic tolerance [3]. This model experiment showed that a drop in ATP is sufficient to produce persisters. We now show that naturally formed persisters are indeed cells with low ATP. This is consistent with stochastic variations in expression of Krebs cycle enzymes producing rare persister cells with low ATP. Taken together, these experiments suggest causality between low ATP and formation of drug tolerant persisters. This notion is consistent with several studies showing that cell metabolism is inherently stochastic and a source of phenotypic heterogeneity [36–38]. There is also a considerable body of literature pointing to a link between persister formation, metabolism, and nutrient depletion [39–43]. In agreement with this, antibiotic efficacy positively correlates with metabolic activity in the cell [44,45]. Interestingly, it has also been shown that in the infection environment, interactions of *S*. *aureus* with other pathogens or with the host immune system can both impact its metabolic activity and induce a decrease in ATP level and an increase in antibiotic tolerance [46–48].

While metabolic activity will determine the level of ATP, it is probably not the sole cause for low target activity. Indeed, our analysis shows that some persisters have an ATP level which is similar to some regular cells that later die (Fig 3D). It appears that there are 2 types of mechanisms which produce persisters—specialized, such as the TisB toxin in *E*. *coli* or the TacT toxins in *Salmonella* [49,50], and a general, low energy level mechanism. Unlike specialized pathways, the energy level mechanism may be operating in many, if not all, bacterial species. Indeed, gene expression is subject to noise [51], and a large population will likely produce rare cells deficient in energy-producing components, which become persisters that survive antibiotic treatment. The low energy level mechanism explains why it is so hard to eradicate persisters both in vitro and in chronic infections. Currently available antibiotics require active, energy-dependent targets in order to kill the cell [52]. Compounds for killing persisters would therefore have to attack targets that do not require energy. The first example of such a compound is acyldepsipeptide (ADEP) which dysregulates the ClpP protease, transforming it into a nonspecific protein hydrolase in an ATP-independent fashion [53], and forces both regular cells and persisters to self-digest [54]. Hydrolysis does not require energy, and other energy-independent hydrolases may be similarly exploited for the development of anti-persister compounds to combat chronic infections. Interestingly, it is also possible to eliminate persisters with antibiotics that do not kill tolerant cells. We recently showed that pulse-dosing oxacillin can effectively treat a biofilm of *S*. *aureus* in vitro [55]. After a first dose, the antibiotic is washed away, allowing persisters to resuscitate, and the second dose is added before they have a chance to restore the biofilm population. Several pulse-dose cycles can then eradicate the biofilm. The significance of understanding the nature of drug tolerance and developing anti-persister approaches is especially important in light of recent reports showing that antibiotic tolerance can favor development of classical resistance [56–58].

## Materials and methods

### Bacterial strains and growth conditions

The strains and plasmids used in this study are listed in S1 Table, and the primers in S2 and S3 Tables. Bacteria were routinely grown at 37°C with aeration at 225 rpm. The media used were LB, MOPS minimum medium supplemented with 0.4% sodium pyruvate or sodium acetate, or EZRD medium as specified in each figure. Ampicillin (100 μg/mL at 30°C) was used for plasmid selection and plasmid maintenance. Arabinose (1 mM or 10 mM) was used for P_*araB*_ induction. Chloramphenicol (25 μg/mL) and kanamycin (50 μg/mL) were used for chromosomal markers selection. The *E*. *coli* backgrounds used in this study were MG1655, MG1655-SB1 (MG1655 CGSC#: 6300, a non-motile variant of MG1655), and W3110D.

### Construction of strains

*aceA* and *icd*, *gltA*, and *sucA* deletion strains were constructed in *E*. *coli* MG1655 via P1 transduction from the Keio collection [59].

*mVenus* translational fusions were constructed by λ Red recombination [59,60] as schematized in S9 Fig.

To construct the chromosomal insertion of iATPSnFr^1.0^, the gene + its RBS in pRset were amplified from the original pRset-*iATPSnFr*^*1*.*0*^ plasmid [24] and cloned into the *Not*I/*Xho*I sites of an in-house modified version of pGRG25 [61], between the *J23100* promoter from the Anderson library (http://parts.igem.org/Promoters/Catalog/Anderson) and the *rrnB(T1)* terminator. The plasmid constructed was used to transform the *E*. *coli* strains of interest, and the next subsequent steps for integration into the chromosome (*attTn7* site) were done according to [61].

Primers used for strain construction or validation are specified in S2 and S3 Tables. Mutations and plasmids were confirmed by PCR and sequencing.

### ATP quantification

Bacteria were inoculated at 1:100 into LB medium from an overnight culture (approximately 16 hours). Cultures were grown at 37°C for approximately 2 hours, before to be treated or not with arsenate. Cultures were washed with PBS, and intracellular ATP concentration of the bulk culture was measured by firefly luciferase using a BacTiter Glo kit (Promega, Madison, WI, USA) according to the manufacturer’s instructions. Bioluminescence RLU were read using a Synergy H1 microplate reader with Gen5 software (Biotek Instruments, Winooski, VT, USA) and were normalized to OD_600_.

### Antibiotic survival assay

Bacteria were inoculated at 1:100 into LB medium from an overnight culture (approximately 16 hours). Cultures were grown at 37°C for 2 to 2.5 hours to reach approximately the same colony forming unit (CFU) number before to be challenged with ciprofloxacin (1 μg/mL) for 5 hours. For CFU count, cultures were washed with NaCl, serially diluted, and plated onto LB agar.

### Growth measurements

Bacteria were grown overnight in LB medium and were inoculated at 1:100 or 1:1,000 into 200 μL of LB or MOPS minimum media supplemented with 0.4% sodium pyruvate or sodium acetate in 96-well microtiter plates. For growth in minimum medium, samples were washed first to remove LB before inoculation. Plates were incubated at 37°C with constant shaking, and OD_600_ was measured using a Synergy H1 microplate reader (BioTek) and Gen5 software (Biotek).

### Flow cytometry analysis and cell sorting

The fluorescent protein levels were analyzed with a BD FACS Aria II flow cytometer (BD Biosciences, San Jose, CA, USA) with a 70-μm nozzle. The cell populations were detected using forward and side scatter (FSC-H and SSC-H) parameters, and gates were created to exclude cell debris. Fluorescence was analyzed with a 488-nm laser and bandpass filter of 545/30 nm. Data were acquired using FACSDiva (BD), and graphs were generated with FlowJo (BD). For survival sorting experiments, exponentially growing cells of W3110D carrying a translational *mVenus* fusion of *aceA*, *icd*, *gltA*, *sucA* (grown in LB at 37°C) were exposed to 1 μg/mL of ciprofloxacin for 4 hours. One hundred and/or 1,000 events per spot were then collected from each fraction (Dim as approximately 5% of the whole population, Middle as approximately 20%, and Bright as approximately 5%) and dispensed directly onto LB agar plates (32 spots per fraction within 1 experiment). The % survival was calculated as the % of the CFU number divided by the total number of events (= cells) sorted onto the plate.

### Confocal microscopy and image analysis

Bacteria expressing iATPSnFr^1.0^ were routinely cultured in LB and treated when indicated 30 minutes with arsenate. Samples were washed with PBS, placed on top of a pad made of 1.5% low melting agarose diluted in PBS, and observed under a ZEISS LSM 710 confocal microscope using 63x oil immersion objective lens. The 2 fluorescent signals 405ex and 488ex were sequentially collected. Differential interference contrast (DIC) image was recorded alongside the 405ex acquisition. Images were acquired by Zen software at a resolution of 1024 × 1024 and lane average of 8 and processed with the Fiji software [62]. For iATPSnFr^1.0^, the 488ex/405ex is indicative of ATP level [24]. For visual representation of the ratios, the Ratio Plus plugin or Image Calculator were used. For quantification of fluorescence, channels were realigned by using the MultiStackReg plugin, background was subtracted (rolling ball radius of 50 pixels), and images were smoothed (3 × 3 pixels) for each of the 2 fluorescence channels. Segmentation analysis was performed on the 405ex channel with MicrobeJ plugins [63], and the ratio 488ex/405ex was calculated by dividing the mean fluorescence of 1 channel by the other for each cell. Due to the difference in optics between confocal microscopy and epifluorescence microscopy, the absolute ratiometric values 488ex/405ex are different in experiments performed with the ZEISS LSM 710 or the Nikon Ti2E.

### Mother machine experiments

#### Mother machine chip preparation

Mother machine chips were made from bonding a PDMS mold of a silicon wafer with a 20 × 50 mm coverslip. Construction of the silicon wafer with the features for the mother machine is described elsewhere [27]. Dimethyl siloxane monomer (Sylgard 184) was mixed in a 10:1 ratio with curing agent, defoamed, and then poured onto the silicon wafer to make the PDMS mold, which was then degassed in vacuum for 1 hour, and then cured at 95°C for another 1 hour. Each mold contains 6 mother machine chips. Individual mother machine chips were cut out from the mold and holes for inlet and outlet were created using a biopsy puncher (0.75 mm). The chips were cleaned by sonication in isopropanol for 30 minutes and were dried with an air-gun and then baked at 95°C for 30 minutes. Similarly, the coverslips were cleaned by sonication in 1M KOH for 20 minutes, followed by sonication in DI water for another 20 minutes. The cleaned coverslips were then dried with an air-gun and then baked for 30 minutes at 95°C. The feature side of the mother machine chips were bonded to the coverslips using oxygen plasma treatment (45 seconds at 55 W and O_2_ pressure set to 170 mTorr) followed by incubation at 95°C for at least 1 hour. Chips were bonded the day before being used in experiments.

#### Loading cells in the mother machine chips

We used a pair of gel-loading tips for loading cells into the feeding lanes and centrifuged the chips to load the cells into the trenches that are orthogonal to the loading lanes. The stationary culture is concentrated 10× before loading into the device. The loaded chip is then spun at 500 rcf in a benchtop centrifuge for 3 minutes, using a custom-built holder, and then flip vertically and spun at 500 rcf for 30 seconds. The centrifugal force rapidly loads the cells in the trenches on both sides of the feeding channel. After loading and connecting the chip to the pumps, left over culture from loading is promptly washed away before the start of imaging acquisition.

#### Microscopy and image acquisition

The mother machine chip loaded with cells was placed on a stage adapter to be placed in a Nikon Ti2E automated stage that enables rapid automated scanning of the chip for collecting data from multiple fields of view. We have optimized the microscope configuration for fast acquisition [27] that enables us to record images from up to 500 fields of views every 5 minutes. Spectra-X light engine from Lumencor was used as the light source to excite the sample, and images were acquired with a sCMOS camera (ANDOR zyla 4.2), through a high NA objective (Apochromat, 40x, 0.95 NA). For the iATPSnFr^1.0^ reporter, images were acquired using the FITC emission filter, with 1 round of excitation at 470 nm with 500 mseconds exposure time and another round at 395 nm with 500 mseconds exposure time. For convenience in the manuscript, we refer to those measurements as 488ex and 405ex, respectively. The entire setup was housed in a temperature-controlled incubator (OKO lab) set at 37°C. Focal drift from minor thermal fluctuations were eliminated by the Nikon Perfect Focus system. The data acquisition was controlled by the Nikon elements software. The data were saved in ND2 format and eventually extracted for analysis into single TIFF files using the analysis software provided by Nikon. The cell size study was done similarly but used phase contrast images acquired with a 100X Oil PH3 objective. The autofluorescence from the WT strain (MG1655-SB1) has been determined negligible in our measurements based on the following average values: MG1655-SB1: 869.62 (405ex) / 670.34 (488ex) in stationary phase (488ex/405ex of 0.77), 695.48 (405ex) / 544.85 (488ex) in exponential phase (488ex/405ex of 0.78); MG1655-SB1-*iATPSnFr*^*1*.*0*^: 1210.20 (405ex) / 987.28 (488ex) in stationary phase (488ex/405ex of 0.82), 1920.00 (405ex) / 2188.30 (488ex) in exponential phase (488ex/405ex of 1.14). Photobleaching and photoactivation effects have also been examined for the iATPSnFr^1.0^ sensor in conditions of acquisition relevant to the experiments performed in this work and have been determined negligible and not impacting the measure of the 488ex/405ex ratio (S10 Fig). In this experiment, stationary cells were loaded in the chip, and the signal from both channels, as well as the ratio reflecting ATP was monitored over time (S10 Fig). As the culture starts recovering from stationary phase (starting from frame 1), protein synthesis will increase, producing more of the sensor, and increased signal from both channels, as well as increase in the ratio, showing increase in ATP levels. We observed that during balanced growth (from frame 7), there is a slight decrease in the intensity of each channel (405ex and 488ex). This could correspond to either a small photobleaching in each channel or a decrease in the total amount of the iATPSnFr^1.0^ protein. Importantly, this affects both channels in a similar way and therefore does not affect the ratio. Neither do we see an increase in the intensity of one of the channels, which rules out a possible photoactivation effect. Careful examinations of those effects would have to be done with other experimental conditions.

#### Data processing

We used a custom-designed image-processing pipeline to process the TIFF for extracting single-cell time series for the mother cells in every trench. The details of the analysis algorithm and software are described in our previous work [27]. Briefly, the pipeline involves 4 major steps: cell segmentation, fluorescence value extraction, single-cell tracking, and trace cleaning. Single cells were segmented from individual TIFFs, and the corresponding binary masks were used to extract intensity values for the fluorescent channels for individual masks. Single-cell tracking and analysis were conducted using a custom-designed scripts (FIJI and MATLAB). More precisely, a clustering-based approach was used to identify the same cells through the TIFFs from consecutive time points and was connected to create time series for individual mother cells. Since the segmentation algorithm is not perfect, we have defined certain criteria to clean up the traces afterwards. Statistical analysis of the results was performed using custom MATLAB scripts. To identify persisters, data sets were scanned for the presence of cells surviving ampicillin treatment. For Fig 3, in the field of view of the positions where survivors were found, the survivor was analyzed as well as all surrounding normal cells over the course of the experiment. For S8 Fig, the normal cells have been analyzed from one entire lane of the mother machine chip for the first time frame of the experiment, regardless of their position with the persisters. The ratio persisters/normal cells doesn’t thus necessarily reflect the survival ratio of the population. The difference between the number of persisters found in both figures (16 in Fig 3 and 5 in S8 Fig) likely reflects the cell loading capacity from one chip to another. Due to the difference in optics between confocal microscopy and epifluorescence microscopy, the absolute ratiometric values 488ex/405ex are different in experiments performed with the ZEISS LSM 710 or the Nikon Ti2E.

#### Statistics

Statistical analyses were performed using Prism 8 (GraphPad software), with two-way ANOVA/Tukey’s multiple comparison to compare Middle and Dim populations in the cell sorting experiment; one-way ANOVA/Tukey’s multiple comparison and Kruskal–Wallis/Dunn’s multiple comparison to assess the effect of arsenate treatment on ATP, in bulk of the population or in single cells, respectively; one-way ANOVA/Tukey’s multiple comparison and Kruskal–Wallis/Dunn’s multiple comparison to compare ATP levels in MG1655 WT, Δ*aceA* and Δ*icd*, in bulk of the population or in single cells, respectively; two-tailed unpaired Student *t* test and two-tailed unpaired Mann–Whitney *U* test, to compare ATP levels in exponential and stationary phase cultures of MG1655 WT, in bulk of the population or in single cells, respectively. Correlation between 2 sets of values was examined by Pearson’s correlation. The *P* values used to determine significance, as well as the meaning of error bars, are indicated in the figures and their legends. Experiments were replicated with reproducible results as indicated in the figure legends. Data exclusion due to technical issues has been realized in the pyruvate/acetate growth assays by excluding data set from abnormal wells. For the persister assay, 2 data points (within 2 different sets of 9 biological replicates) showed seemingly abnormal values, have been identified as outlier using GraphPad Prism and Grubbs’ test (alpha 0.01), and were removed from the analysis.

## Supporting information

S1 Fig Impaired metabolism leads to an increase in antibiotics survival. (A) *E*. *coli* MG1655 WT, Δ*aceA*, and Δ*icd* strains were grown 2 hours to 2.5 hours in LB at 37°C to exponential phase and challenged with ciprofloxacin (1 μg/mL). Survival has been assessed by culture plating and CFU counting. Data are the average results from 3 independent experiments performed with 3 biological replicates (*n =* 8 or 9). Error bars represent standard deviations. (B) Distribution of the iATPSnFr^1.0^ 488ex/405ex ratio per single cell measured by microscopy in exponential cultures of MG1655, Δ*aceA*, and Δ*icd* strains grown in LB at 37°C. Thick lanes represent the median, and secondary lanes the quartiles. Significance was determined using Kruskal–Wallis and Dunn’s multiple comparison tests. Observations were performed with a confocal microscope. (C) Bulk ATP levels were measured in MG1655, Δ*aceA*, and Δ*icd* strains grown in LB 37°C with firefly luciferase assay and normalized with OD_600_. Data are the average results from 3 independent experiments performed with 3 biological replicates (*n* = 9). Error bars represent standard deviations. Significance was determined using one-way ANOVA and Tukey’s multiple comparison tests. The underlying data for this figure can be found in S1 Data.
(TIF)

S2 Fig Growth of the fluorescent reporter strains. MG1655 Krebs cycle KO strains and W3110D Krebs cycle *mVenus* fusion strains were grown in LB (A), in MOPS minimum medium with 0.4% sodium pyruvate (B), or 0.4% sodium acetate (C) as sole carbon source at 37°C. (D) MG1655-SB1 strains expressing or not *iATPSnFr*^*1*.*0*^ were grown in LB at 37°C. In (A), (B), (C), and (D), each lane represents a biological replicate coming from experiments performed 3 times (*n* > 6). The underlying data for this figure can be found in S1 Data.
(TIF)

S3 Fig Fluorescence analysis of the *mVenus* fusions. Representative histogram of fluorescence of the *mVenus* fusions before (A) and after (B) ciprofloxacin treatment of W3110D: black lane, *aceA-mVenus*: red-lane, *icd-mVenus*: blue lane, *gltA-mVenus*: green lane, *sucA-mVenus*: orange lane. For each of those fusions, (C), (D), (E), (F), respectively, show the histogram of fluorescence before (black lane) and after (red lane) ciprofloxacin treatment for each fusion (left panel), and the post-sorting purity analysis of the Dim (light grey), Middle (grey), and Bright (dark grey) sorted fractions (right panel). The number of events represented in the *y* axis is normalized for each graph. The underlying data for this figure can be found in S1 Data.
(TIF)

S4 Fig Reporting ATP in exponential and stationary phase cultures at the single-cell level. (A) Representative images of exponential (upper panel) and stationary (lower panel) phase cultures of MG1655_*iATPSnFr*^*1*.*0*^ cultured in LB at 37°C. The fluorescent signals 405ex and 488ex are false colored in magenta and green, respectively. In the ratiometric 488ex/405ex panel, orange/yellow cells correspond to cells with higher ATP, and blue cells with lower ATP. Scale bar, 5 μm. White arrow shows the presence of a low 488ex/405ex ratio cell within exponential culture. Observations were performed with a confocal microscope. (B) Distribution of the iATPSnFr^1.0^ 488ex/405ex ratio in exponential and stationary phase cultures. Thick lanes represent the median, and secondary lanes the quartiles. Significance was determined using two-tailed unpaired Mann–Whitney *U* test. Data are representative of experiments made twice giving similar results. (C) Bulk ATP levels were measured in MG1655 strain grown in the same conditions as in (A) with firefly luciferase assay and normalized with OD_600_. Data are the average results from 2 independent experiments performed with 3 biological replicates (*n =* 6). Error bars represent standard deviations. Significance was determined by using two-tailed unpaired Student *t* test. The underlying data for this figure can be found in S1 Data.
(TIF)

S5 Fig Kymographs of the different phenotypes observed. The fluorescent panels represent the addition of iATPSnFr^1.0^ 405ex and 488ex intensities. Observations were performed with a time-lapse epifluorescence microscope.
(TIF)

S6 Fig Kymographs of the persisters analyzed. (A) Left panel: ATP value at the beginning of the experiment in persisters. Data represent the single-cell 488ex/405ex ratio values analyzed for each of the 16 persisters at the first time frame in Fig 3D histogram. The black horizontal dashed line represents the mean 488ex/405ex ratio of the normal cells (0.6303). Right panel: Kymographs of the 16 persisters analyzed in Fig 3C. The iATPSnFr^1.0^ ratiometric 488ex/405ex panels represent ATP level. The color code has been scaled individually for each of the 16 kymographs. Persister number 2 harbors a slow growth pattern different than the others which don’t grow in presence of ampicillin. The white rectangles indicate the time frame where the first septal invagination of each persister is observed (time before first division). Observations were performed with a time-lapse epifluorescence microscope. (B) For each persister, time before first division is plotted over the iATPSnFr^1.0^ 488ex/405ex ratio at the first time frame of the time lapse (t = 1). Correlation was examined by Pearson’s correlation. The underlying data for this figure can be found in S1 Data.
(TIF)

S7 Fig iATPSnFr^1.0^ 488ex/405ex ratio in the mother machine. (A) Each data point represents the iATPSnFr^1.0^ 488ex/405ex ratio per single cell over time frames (frames interval is 30 minutes) for normal cells (in black) and persister cells (in red). (B) Distribution of the iATPSnFr^1.0^ 488ex/405ex ratio per single cell for normal cells (in black) and persister cells (in red) according to their position in the mother machine, at the first time frame of the time lapse (t = 1), and (C) immediately before the antibiotic is added (t = 5). Observations were performed with a time-lapse epifluorescence microscope. The underlying data for this figure can be found in S1 Data.
(TIF)

S8 Fig Persisters, cell size, and ATP. (A) Distribution of cell size area. (B) Distribution of the iATPSnFr^1.0^ 488ex/405ex ratio. A mother machine experiment using the same setup as in Fig 3 was performed. The light grey bars represent all non-persister cells (23,766 cells) at the first time frame, immediately after loading in the mother machine from a stationary phase culture. Dark grey corresponds to non-persister cells with sizes smaller than the average persister cells (5,596 cells). The 5 persisters found in this experiment are plotted in red. Observations were performed with a time-lapse epifluorescence microscope. The underlying data for this figure can be found in S1 Data.
(TIF)

S9 Fig General schematic of the construction of in-frame chromosomal mVenus fusions. Schematic of the construction of the translational *mVenus* fusions. Two-round PCRs were performed. The template used was the pKDN31 plasmid (a gift from Barry Wanner), and the polymerase used was the KOD. Final PCRs were precipitated with ethanol, dried up, then dissolved in H_2_O, before to be used to electrotransform W3110D/pKD46. Electrocompetent cells of W3110D/pKD46 were prepared according to the standard protocol for λ Red recombination with a higher concentration of L-arabinose (10 mM) [59,60]. The double selection was done using resistance to chloramphenicol (25 μg/mL) and sensitivity to ampicillin (100 μg/mL at 30°C), and clones were PCR checked using Left and Down primers (S3 Table). Finally, bar code sequencing was performed after amplification with Left and NYP244 primers (S3 Table).
(TIF)

S10 Fig Evaluation of photobleaching and photoactivation effects for the iATPSnFr^1.0^ sensor. Stationary phase cells were concentrated and loaded in the device, and fresh EZRDM was flowed until cells are in balanced growth (from time frame 7 to the end of the experiment). Frames were taken 30 minutes apart. Changes in the 405ex and 488ex signals of the iATPSnFr^1.0^ sensor (on the right *y* axis) and in the 488ex/405ex ratio (on the left *y* axis) were monitored over time. Data represented are the mean of the iATPSnFr^1.0^ 488ex, 405ex, and 488ex/405ex ratio per single cell over time frames (*n =* 2,670). Error bars represent standard errors. Observations were performed with a time-lapse epifluorescence microscope. The underlying data for this figure can be found in S1 Data.
(TIF)

S1 Table Table of strains. (XLSX)

S2 Table Table of primers (1). (XLSX)

S3 Table Table of primers (2). (XLSX)

S1 Movie Kymographs of a persister and a non-persister cells over time. MG1655-SB1_*iATPSnFr*^*1*.*0*^ stationary phase cells were concentrated and loaded in the device. The culture used to load the device was first flowed in the device for 1 hour (black arrow), following by 1 hour of fresh EZRDM (grey arrow), by EZRDM + ampicillin (50 μg/mL) for 5 hours (red arrow), and finally by fresh EZRDM again (grey arrow). Frames were taken 30 minutes apart. The upper panel represents the outcome of a mother cell that dies following ampicillin treatment. The lower panel represents the outcome of a persister cell that survives ampicillin treatment. The iATPSnFr^1.0^ ratiometric 488ex/405ex panels represent ATP level. Observations were performed with a time-lapse epifluorescence microscope.
(MP4)

S1 Data Numerical raw data. Underlying numerical raw data for Figs 1D, 2C, 2D, 2E, 3C, 3D, 3E, S1A, S1B, S1C, S2A, S2B, S2C, S2D, S3A, S3B, S3C, S3D, S3E, S3F, S4B, S4C, S6B, S7A, S7B, S7C, S8A, S8B and S10.
(XLSX)

## Abbreviations

ADEP: acyldepsipeptide
CFU: colony forming unit
DIC: differential interference contrast
FACS: fluorescence-activated cell sorting
Icd: isocitrate dehydrogenase
TA: toxin-antitoxin
WT: wild type
