# Supplementary figures and images for: Bacterial persisters are a stochastically formed subpopulation of low-energy cells
Source: PLoS Biol. 2021 Apr 19;19(4):e3001194. doi: 10.1371/journal.pbio.3001194 (PMC8084331; doi:10.1371/journal.pbio.3001194)

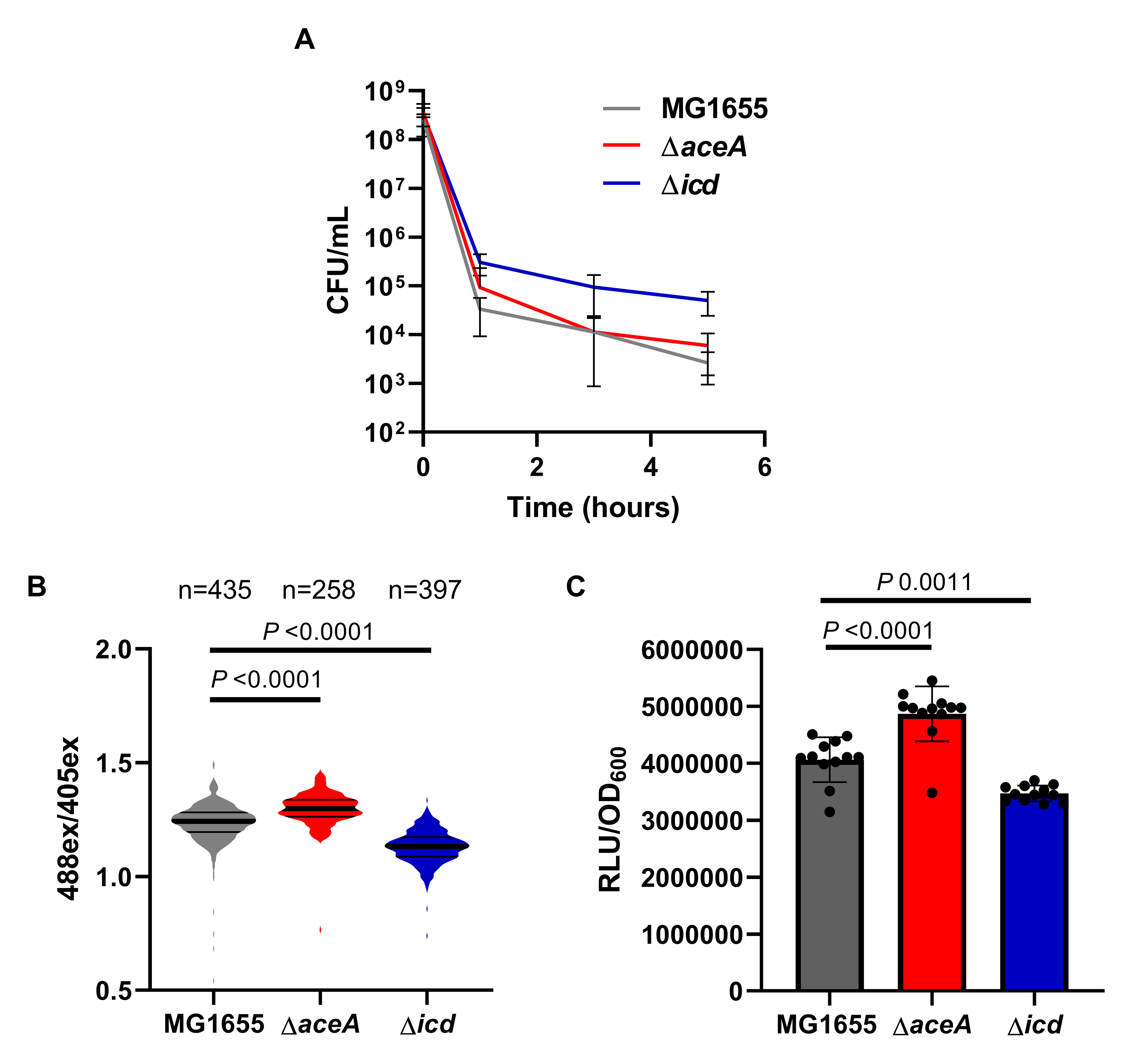

Supplement: S1 Fig — (A) E. coli MG1655 WT, ΔaceA, and Δicd strains were grown 2 hours to 2.5 hours in LB at 37°C to exponential phase and challenged with ciprofloxacin (1 μg/mL). Survival has been assessed by culture plating and CFU counting. Data are the average results from 3 independent experiments performed with 3 biological replicates (n = 8 or 9). Error bars represent standard deviations. (B) Distribution of the iATPSnFr1.0 488ex/405ex ratio per single cell measured by microscopy in exponential cultures of MG1655, ΔaceA, and Δicd strains grown in LB at 37°C. Thick lanes represent the median, and secondary lanes the quartiles. Significance was determined using Kruskal–Wallis and Dunn’s multiple comparison tests. Observations were performed with a confocal microscope. (C) Bulk ATP levels were measured in MG1655, ΔaceA, and Δicd strains grown in LB 37°C with firefly luciferase assay and normalized with OD600. Data are the average results from 3 independent experiments performed with 3 biological replicates (n = 9). Error bars represent standard deviations. Significance was determined using one-way ANOVA and Tukey’s multiple comparison tests. The underlying data for this figure can be found in S1 Data. (TIF) [file pbio.3001194.s001.tif]

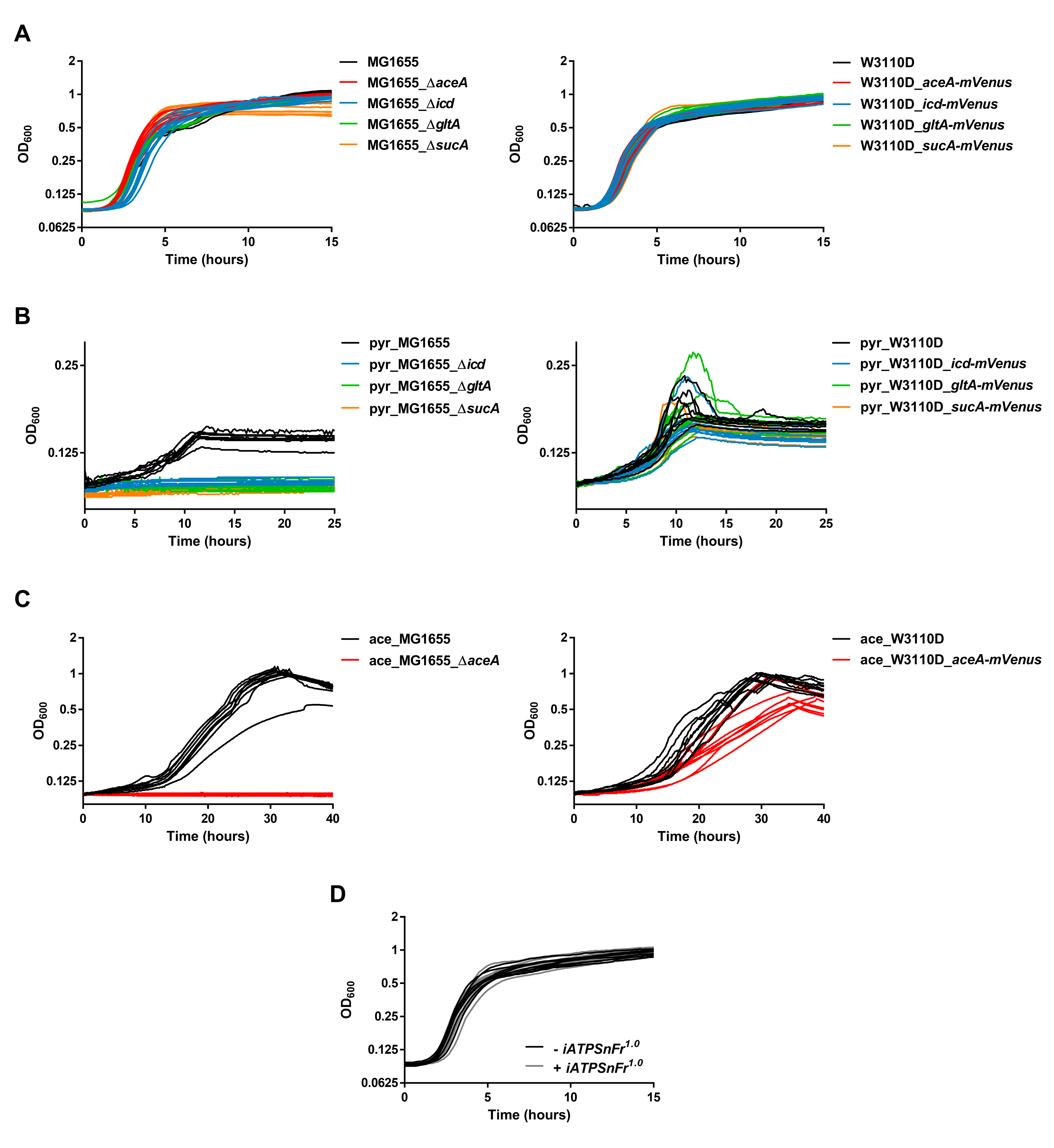

Supplement: S2 Fig — MG1655 Krebs cycle KO strains and W3110D Krebs cycle mVenus fusion strains were grown in LB (A), in MOPS minimum medium with 0.4% sodium pyruvate (B), or 0.4% sodium acetate (C) as sole carbon source at 37°C. (D) MG1655-SB1 strains expressing or not iATPSnFr1.0 were grown in LB at 37°C. In (A), (B), (C), and (D), each lane represents a biological replicate coming from experiments performed 3 times (n > 6). The underlying data for this figure can be found in S1 Data. (TIF) [file pbio.3001194.s002.tif]

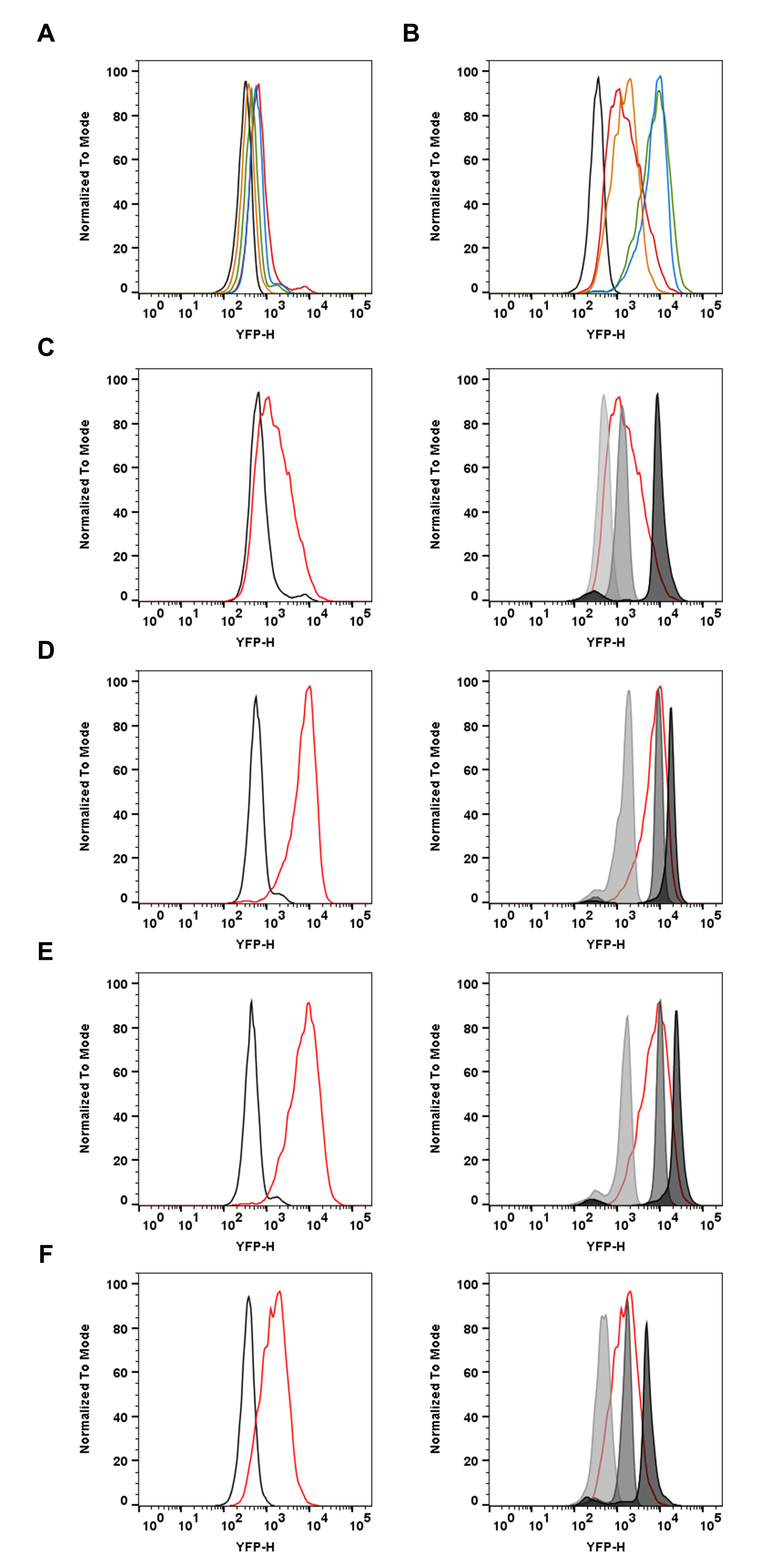

Supplement: S3 Fig — Representative histogram of fluorescence of the mVenus fusions before (A) and after (B) ciprofloxacin treatment of W3110D: black lane, aceA-mVenus: red-lane, icd-mVenus: blue lane, gltA-mVenus: green lane, sucA-mVenus: orange lane. For each of those fusions, (C), (D), (E), (F), respectively, show the histogram of fluorescence before (black lane) and after (red lane) ciprofloxacin treatment for each fusion (left panel), and the post-sorting purity analysis of the Dim (light grey), Middle (grey), and Bright (dark grey) sorted fractions (right panel). The number of events represented in the y axis is normalized for each graph. The underlying data for this figure can be found in S1 Data. (TIF) [file pbio.3001194.s003.tif]

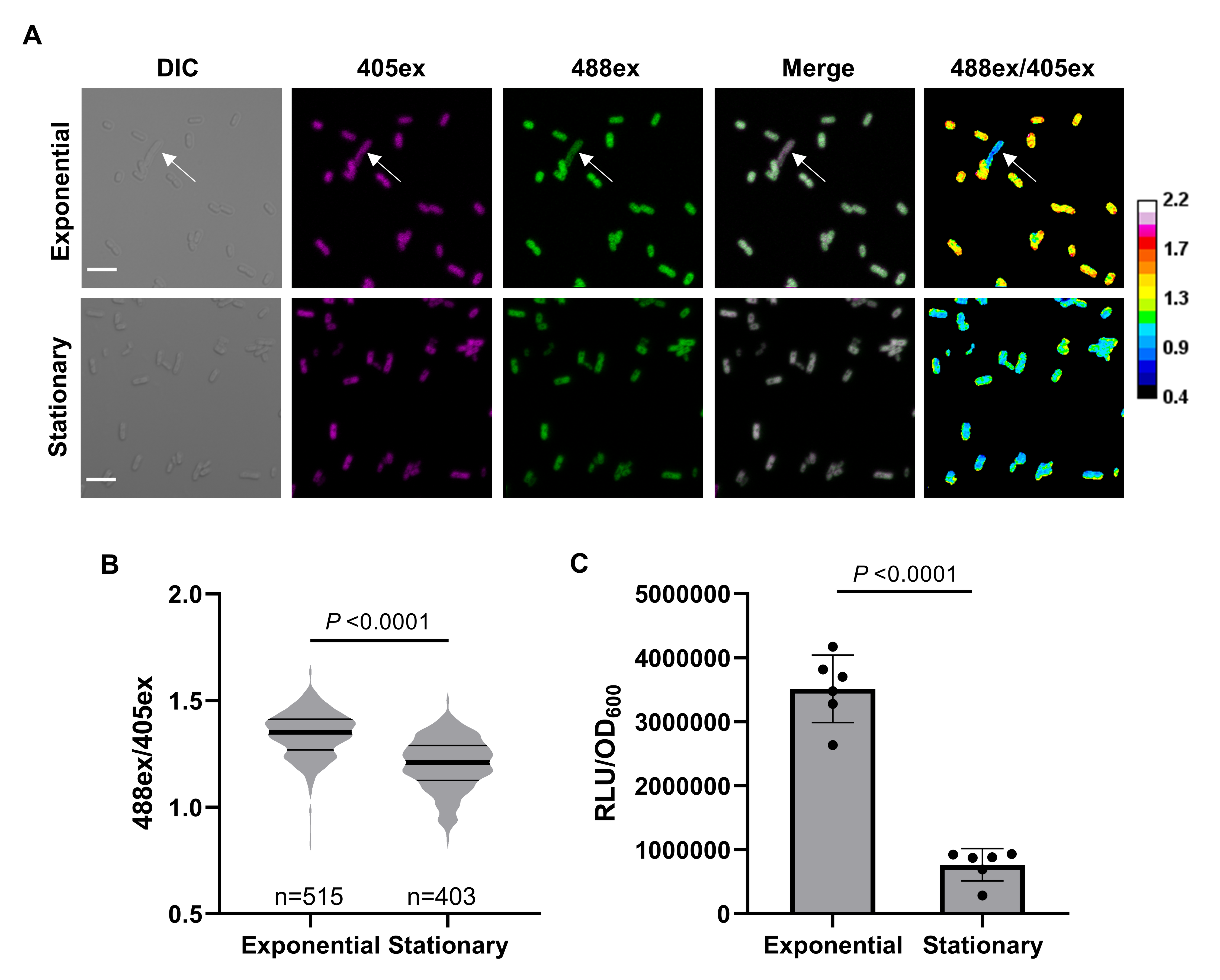

Supplement: S4 Fig — (A) Representative images of exponential (upper panel) and stationary (lower panel) phase cultures of MG1655_iATPSnFr1.0 cultured in LB at 37°C. The fluorescent signals 405ex and 488ex are false colored in magenta and green, respectively. In the ratiometric 488ex/405ex panel, orange/yellow cells correspond to cells with higher ATP, and blue cells with lower ATP. Scale bar, 5 μm. White arrow shows the presence of a low 488ex/405ex ratio cell within exponential culture. Observations were performed with a confocal microscope. (B) Distribution of the iATPSnFr1.0 488ex/405ex ratio in exponential and stationary phase cultures. Thick lanes represent the median, and secondary lanes the quartiles. Significance was determined using two-tailed unpaired Mann–Whitney U test. Data are representative of experiments made twice giving similar results. (C) Bulk ATP levels were measured in MG1655 strain grown in the same conditions as in (A) with firefly luciferase assay and normalized with OD600. Data are the average results from 2 independent experiments performed with 3 biological replicates (n = 6). Error bars represent standard deviations. Significance was determined by using two-tailed unpaired Student t test. The underlying data for this figure can be found in S1 Data. (TIF) [file pbio.3001194.s004.tif]

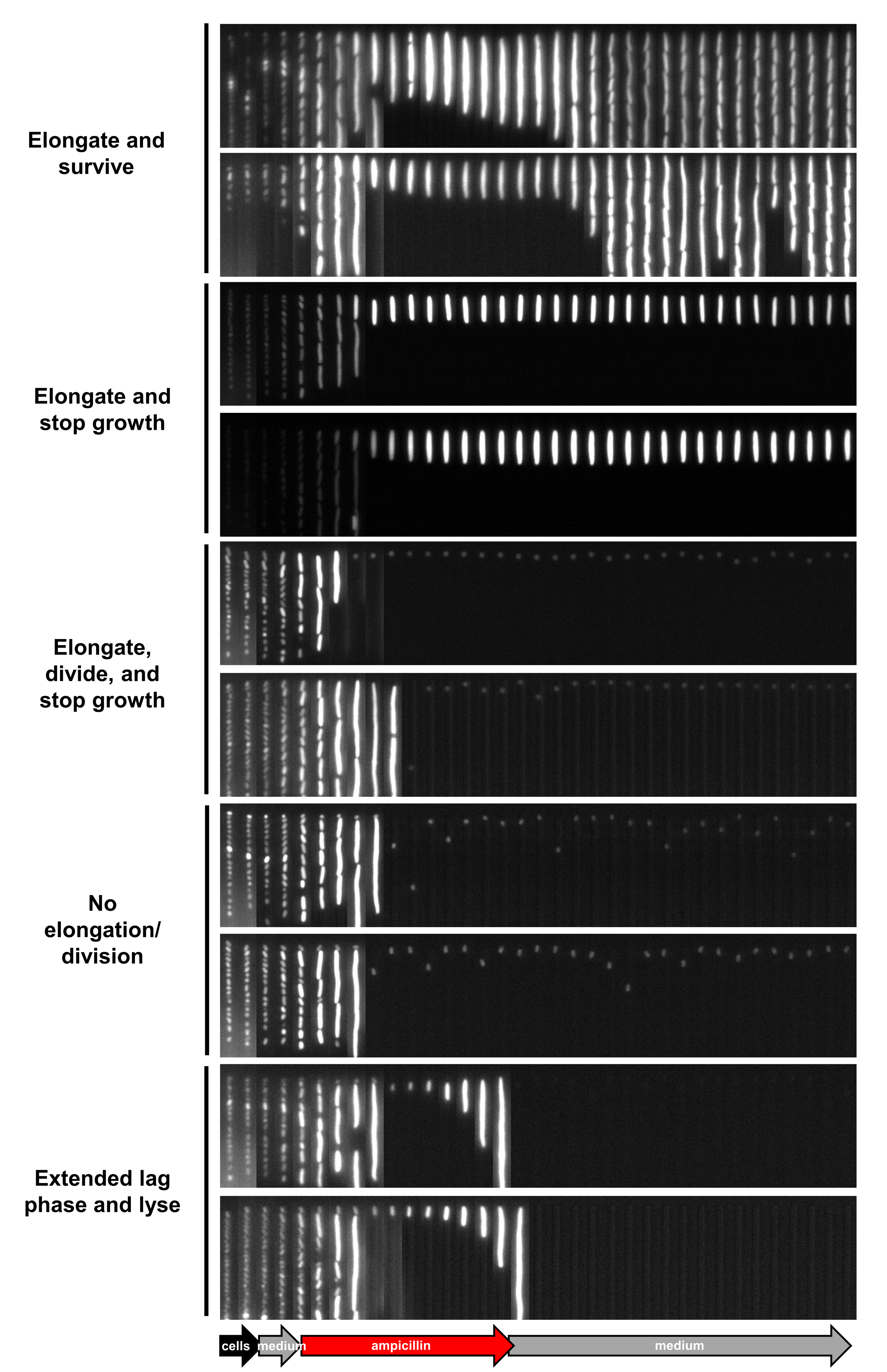

Supplement: S5 Fig — The fluorescent panels represent the addition of iATPSnFr1.0 405ex and 488ex intensities. Observations were performed with a time-lapse epifluorescence microscope. (TIF) [file pbio.3001194.s005.tif]

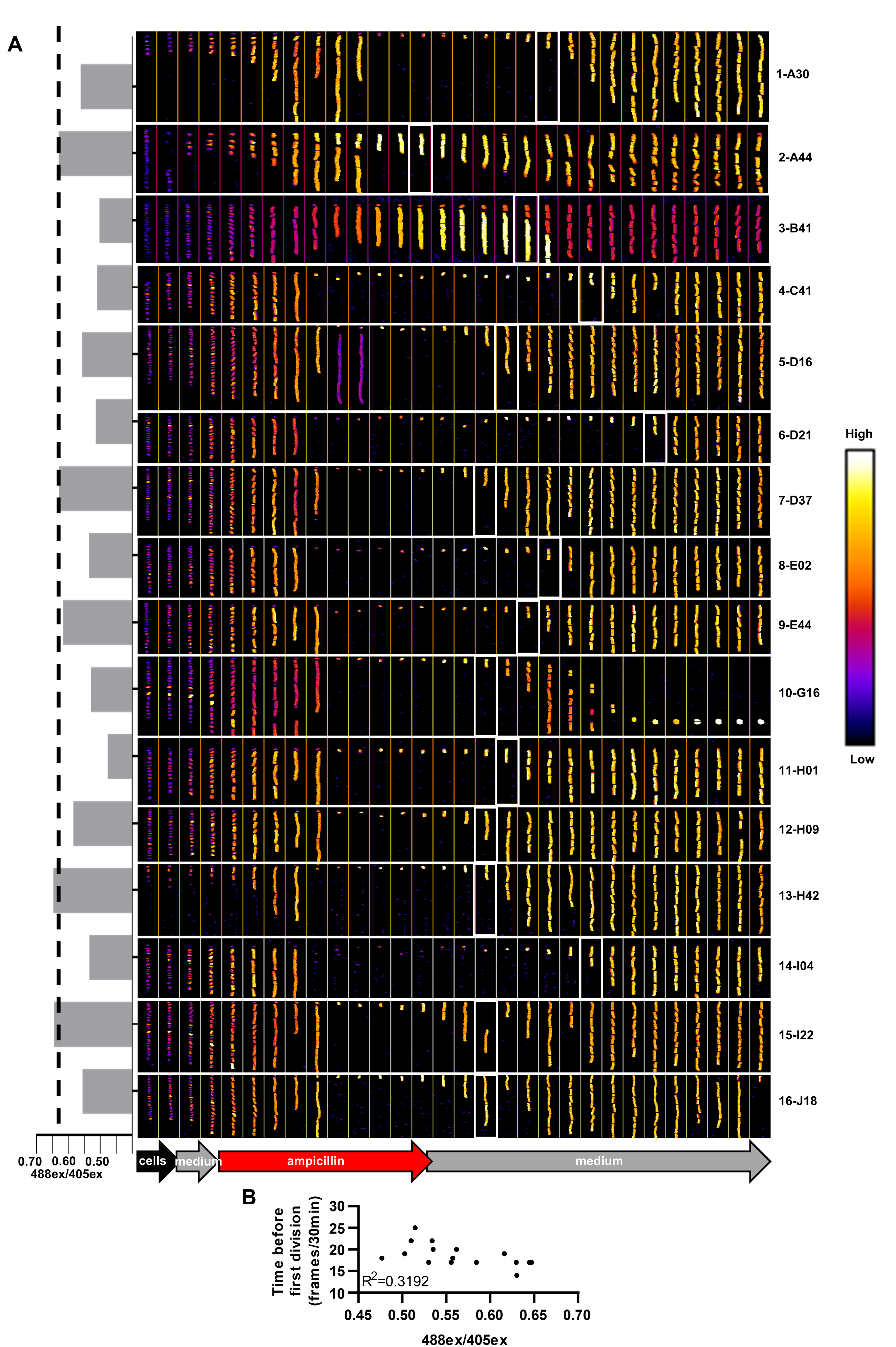

Supplement: S6 Fig — (A) Left panel: ATP value at the beginning of the experiment in persisters. Data represent the single-cell 488ex/405ex ratio values analyzed for each of the 16 persisters at the first time frame in Fig 3D histogram. The black horizontal dashed line represents the mean 488ex/405ex ratio of the normal cells (0.6303). Right panel: Kymographs of the 16 persisters analyzed in Fig 3C. The iATPSnFr1.0 ratiometric 488ex/405ex panels represent ATP level. The color code has been scaled individually for each of the 16 kymographs. Persister number 2 harbors a slow growth pattern different than the others which don’t grow in presence of ampicillin. The white rectangles indicate the time frame where the first septal invagination of each persister is observed (time before first division). Observations were performed with a time-lapse epifluorescence microscope. (B) For each persister, time before first division is plotted over the iATPSnFr1.0 488ex/405ex ratio at the first time frame of the time lapse (t = 1). Correlation was examined by Pearson’s correlation. The underlying data for this figure can be found in S1 Data. (TIF) [file pbio.3001194.s006.tif]

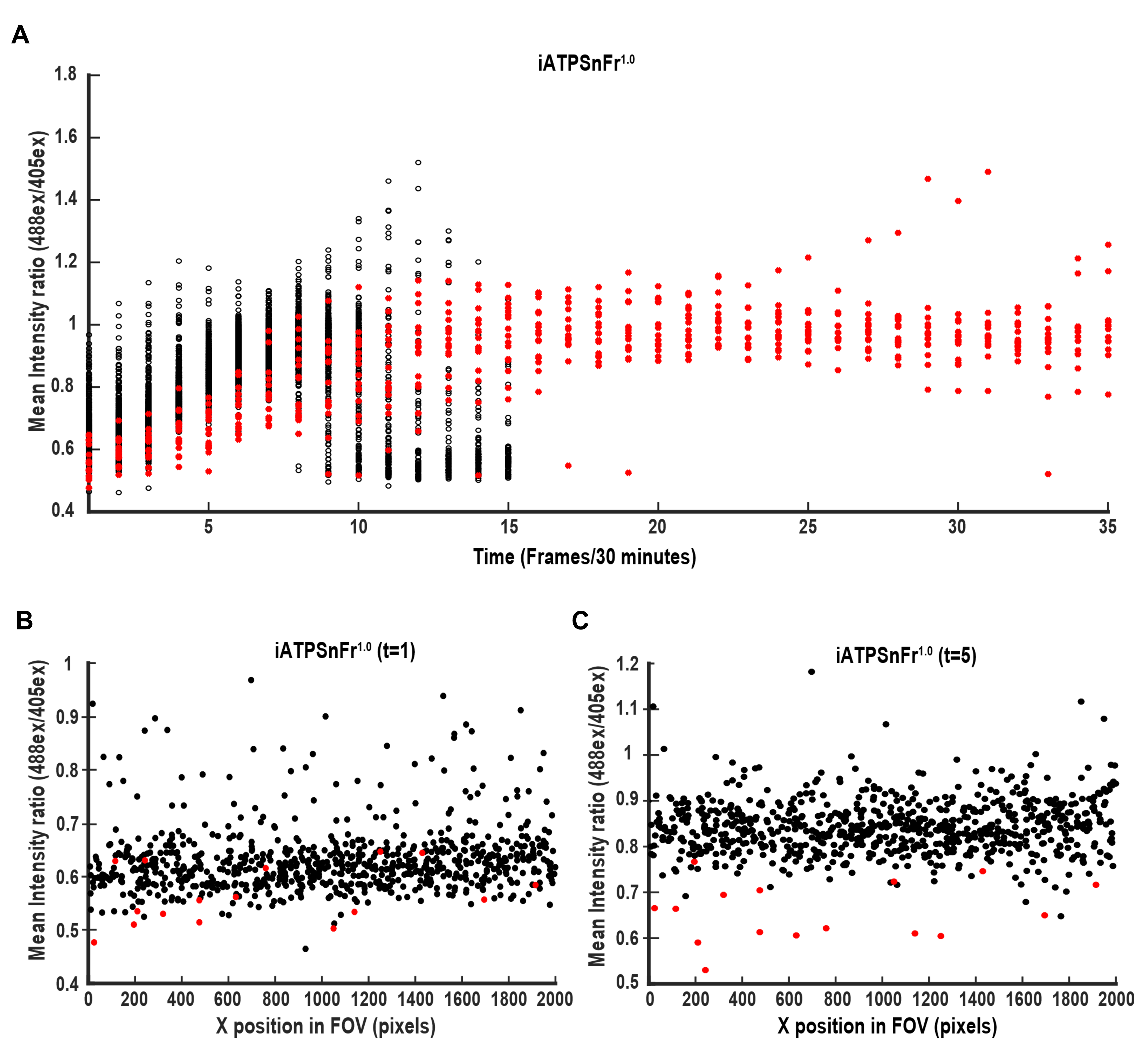

Supplement: S7 Fig — (A) Each data point represents the iATPSnFr1.0 488ex/405ex ratio per single cell over time frames (frames interval is 30 minutes) for normal cells (in black) and persister cells (in red). (B) Distribution of the iATPSnFr1.0 488ex/405ex ratio per single cell for normal cells (in black) and persister cells (in red) according to their position in the mother machine, at the first time frame of the time lapse (t = 1), and (C) immediately before the antibiotic is added (t = 5). Observations were performed with a time-lapse epifluorescence microscope. The underlying data for this figure can be found in S1 Data. (TIF) [file pbio.3001194.s007.tif]

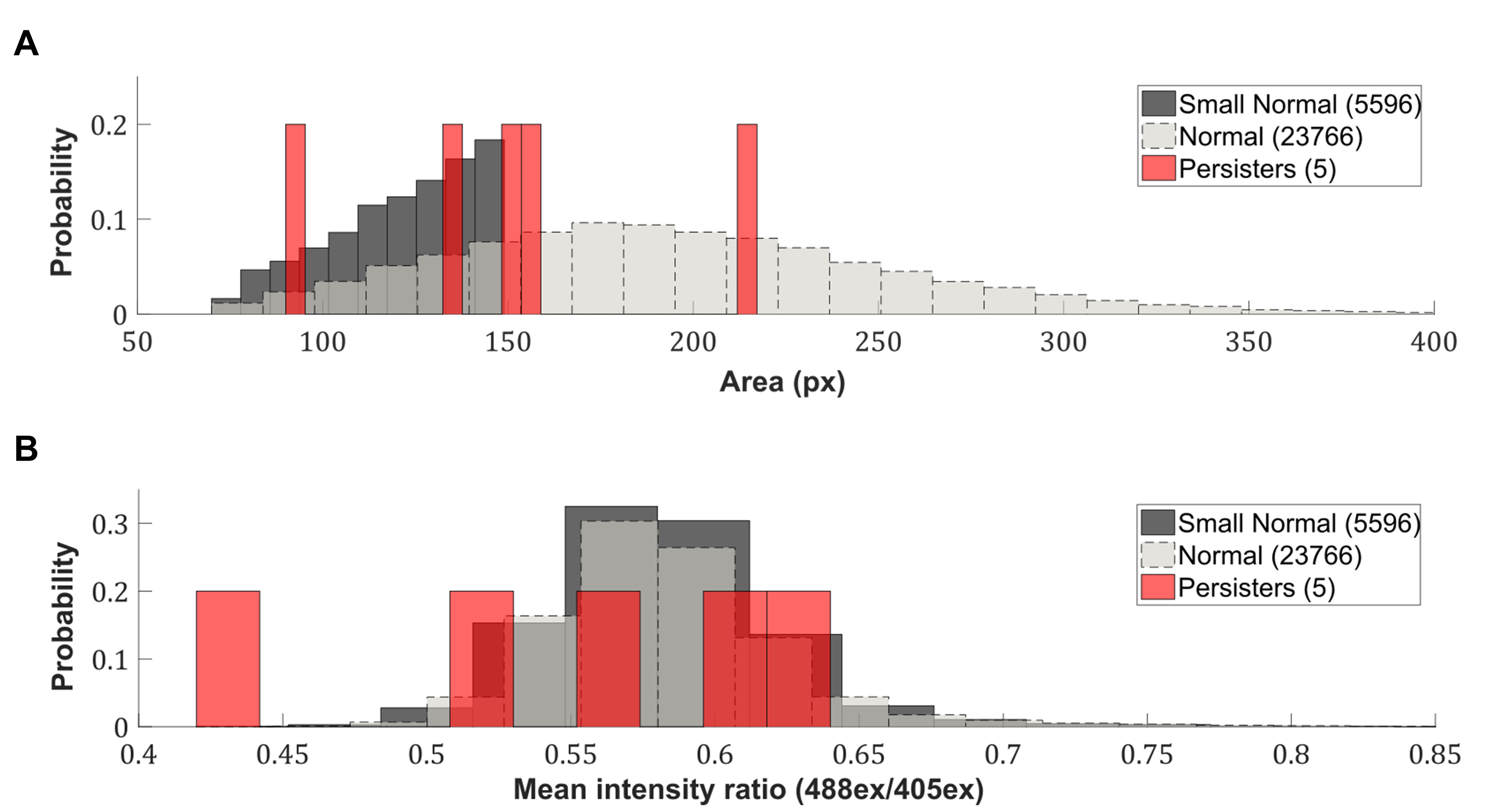

Supplement: S8 Fig — (A) Distribution of cell size area. (B) Distribution of the iATPSnFr1.0 488ex/405ex ratio. A mother machine experiment using the same setup as in Fig 3 was performed. The light grey bars represent all non-persister cells (23,766 cells) at the first time frame, immediately after loading in the mother machine from a stationary phase culture. Dark grey corresponds to non-persister cells with sizes smaller than the average persister cells (5,596 cells). The 5 persisters found in this experiment are plotted in red. Observations were performed with a time-lapse epifluorescence microscope. The underlying data for this figure can be found in S1 Data. (TIF) [file pbio.3001194.s008.tif]

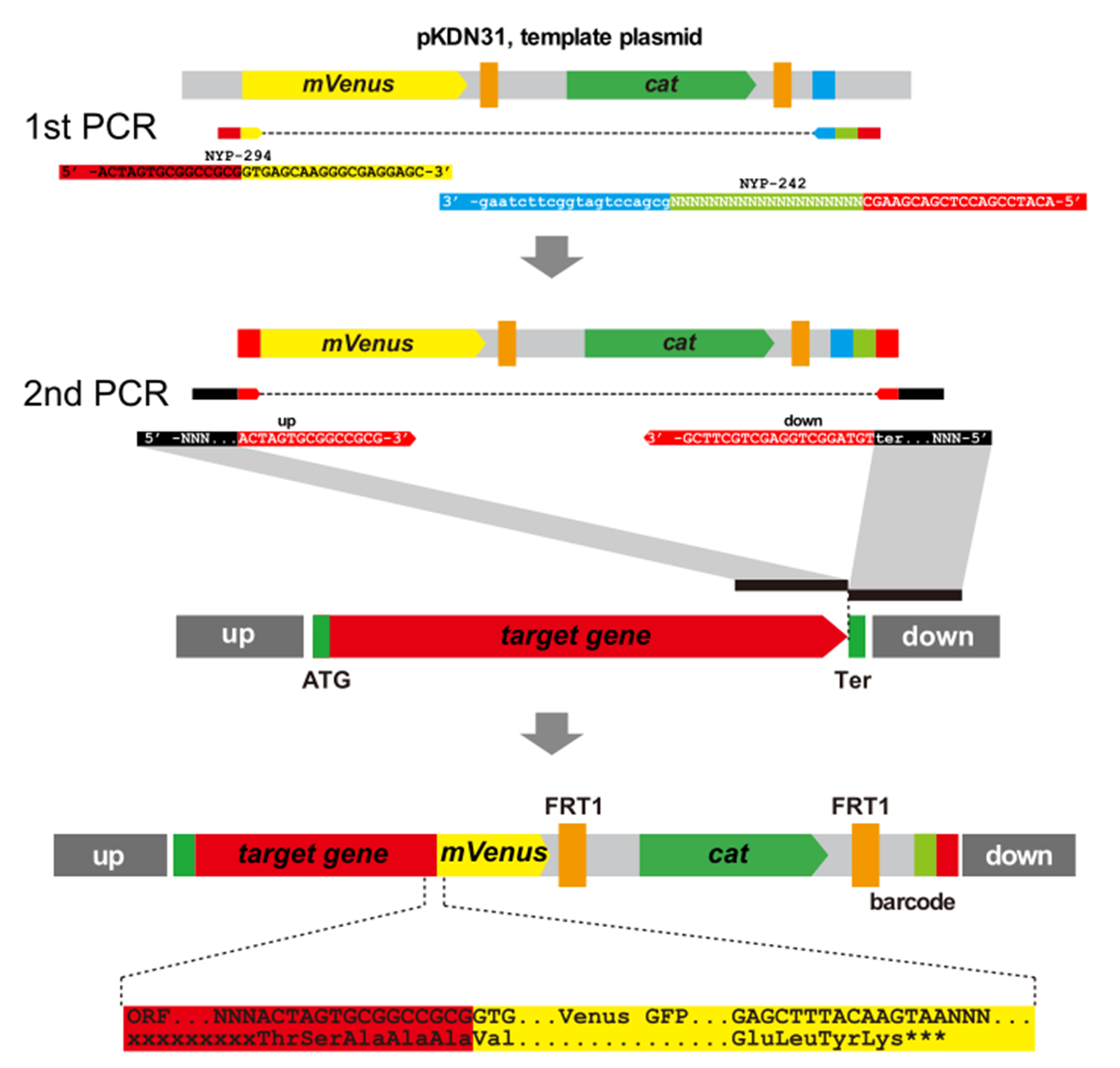

Supplement: S9 Fig — Schematic of the construction of the translational mVenus fusions. Two-round PCRs were performed. The template used was the pKDN31 plasmid (a gift from Barry Wanner), and the polymerase used was the KOD. Final PCRs were precipitated with ethanol, dried up, then dissolved in H2O, before to be used to electrotransform W3110D/pKD46. Electrocompetent cells of W3110D/pKD46 were prepared according to the standard protocol for λ Red recombination with a higher concentration of L-arabinose (10 mM) [59,60]. The double selection was done using resistance to chloramphenicol (25 μg/mL) and sensitivity to ampicillin (100 μg/mL at 30°C), and clones were PCR checked using Left and Down primers (S3 Table). Finally, bar code sequencing was performed after amplification with Left and NYP244 primers (S3 Table). (TIF) [file pbio.3001194.s009.tif]

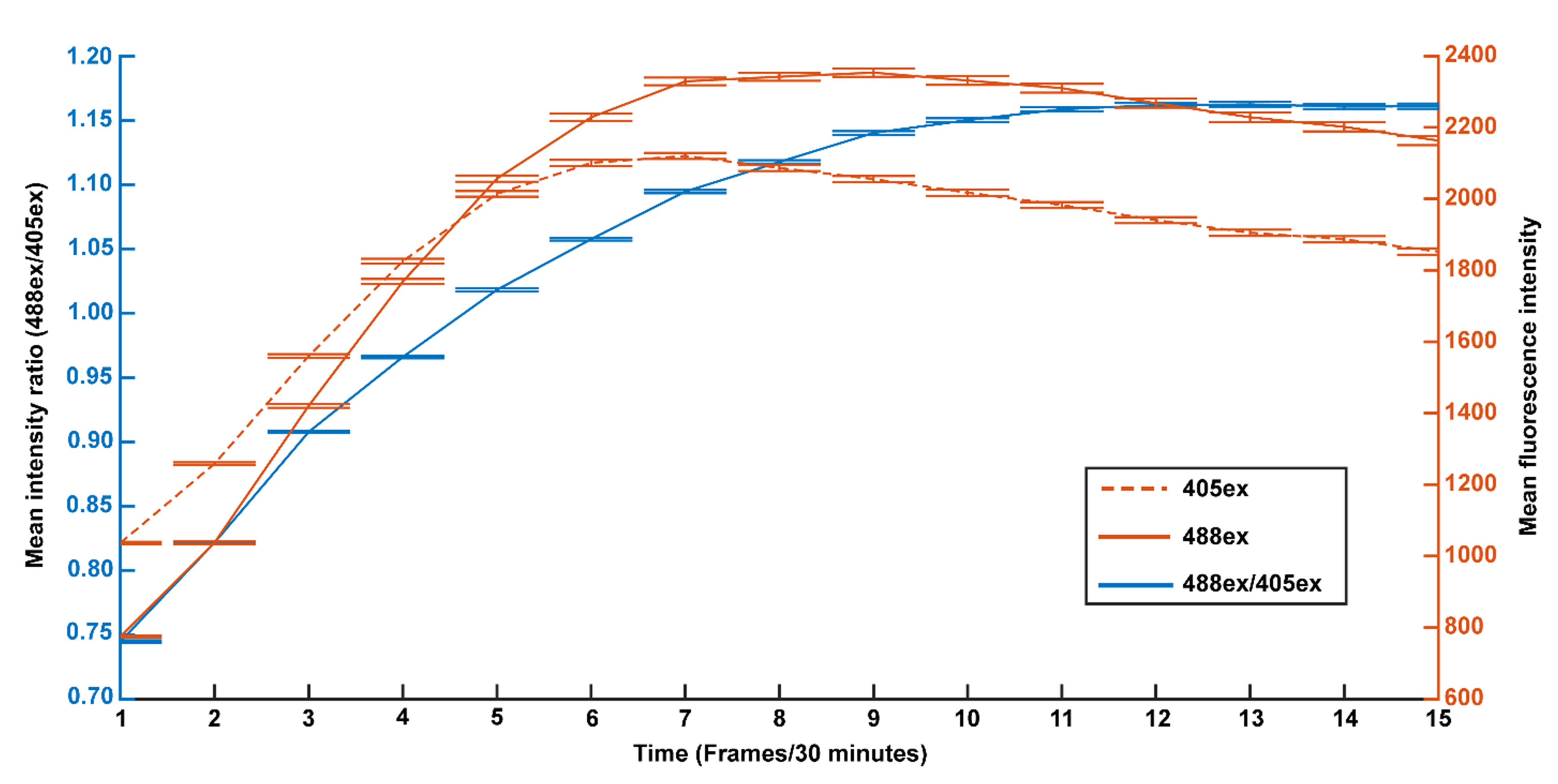

Supplement: S10 Fig — Stationary phase cells were concentrated and loaded in the device, and fresh EZRDM was flowed until cells are in balanced growth (from time frame 7 to the end of the experiment). Frames were taken 30 minutes apart. Changes in the 405ex and 488ex signals of the iATPSnFr1.0 sensor (on the right y axis) and in the 488ex/405ex ratio (on the left y axis) were monitored over time. Data represented are the mean of the iATPSnFr1.0 488ex, 405ex, and 488ex/405ex ratio per single cell over time frames (n = 2,670). Error bars represent standard errors. Observations were performed with a time-lapse epifluorescence microscope. The underlying data for this figure can be found in S1 Data. (TIF) [file pbio.3001194.s010.tif]
